# Antimicrobial Effect Of Visible Blue Light Used In A Minimally Invasive Intramedullary Fracture Stabilization System

**DOI:** 10.7150/jbji.35629

**Published:** 2019-09-18

**Authors:** Guido W. Van Oijen, Peter D. Croughs, Tjebbe Hagenaars, Michael H.J. Verhofstad, Esther M.M. Van Lieshout

**Affiliations:** 1 Trauma Research Unit Department of Surgery, Erasmus MC, University Medical Center Rotterdam, Rotterdam, The Netherlands; 2 Department of Microbiology, Erasmus MC, University Medical Center Rotterdam, Rotterdam, The Netherlands

**Keywords:** Intramedullary, antimicrobial, blue light, fracture stabilization, *S. aureus*

## Abstract

**Introduction:** Since 2009, the IlluminOss^®^ System is being used as an intramedullary fracture treatment. The system is characterized by the use of blue light to polymerize liquid monomer after its infusion in a polyethylene terephthalate balloon. Very few infections of the material have been observed, which might be explained by the possible antimicrobial side-effect of the blue light used in this intramedullary fracture stabilization system. This study aimed to assess this antimicrobial (side-)effect on *S. aureus*.

**Methods:** A suspension of 1.5 x 10^3^ CFU/ml of 8325-4 *S. aureus* was placed into five, custom made, black delrin cylinders. The implant was placed into the cylinders and the light source was activated for 200, 400, 600, 800, or 1,000 seconds. 100 µL of the light exposed suspension was grafted on blood agar and placed in a 35 degrees Celsius incubator for 24 hours. Colonies on each agar plate were counted and compared to the control plates (no blue light exposure).

**Results:** The control plates showed a mean of 85 ± 15 colonies per plate. A statistically significant decrease was observed after 600 seconds of exposure time; mean colony count of 63 ± 4 (p <0.05). The absolute reduction was 24 ± 14 after 600 seconds exposure time. At 800 and 1,000 seconds, no statistically significant reduction was found compared with the control plates (means 72 ± 10 and 83 ± 14 colonies, respectively).

**Conclusions:** In this study only a temporary reduction of *S. aureus* was observed.

If future research regarding the antimicrobial characteristics of blue light used in the IlluminOss^®^ System is desired, it should focus on the need for oxygen and its availability and the dose and manner of applying the light.

## Introduction

The IlluminOss^®^ System is a surgical treatment modality designed to stabilize low-weight bearing longbone fractures. It is commercially available in many countries since 2009. The essence of the system is that a transparent polyethylene terephthalate (PET)-balloon is introduced into the medullary canal of the bone and subsequently inflated by a liquid, transparent monomer. This monomer hardens by exposure to blue light, transmitted to the balloon by a transparent tube 1. During the hardening process, the blue light is visible at the exterior side of the balloon.

The drawback of any surgical implant is that it can be colonized by bacteria, resulting in a biofilm. A biofilm is a first step in the development of an infection. Although the overall infection rate of surgically reduced and internally fixated simple fractures is reasonably low, these infected cases might result in substantial morbidity and costs 2,3. Development of new methods to prevent and/or treat infections are of great importance 4-6. By principle, the implants of the intramedullary fracture stabilization system can be contaminated by bacteria as well. However, because of the use of antimicrobial blue light frequencies, the company theorizes this implant is less at risk for infection than metal implants. Since there are no prospective randomized trials executed yet, no hard evidence for this theory exists.

Inactivation of microorganisms by visible light is an old technology which got new attention in this era of antimicrobial resistance. This technology, known as photodynamic therapy, utilizes exogenous photosensitizers (like phenothiazines and porphyrines), which will produce intracellular reactive oxygen species (ROS) when they become excited by different wavelengths of light in the presence of oxygen. These reactive oxygen species, such as singlet oxygen and hydroxyl radicals, damage nucleic acids and other cellular structures, which in turn results in inactivation and/or apoptosis of living entities such as bacteria 7-9.

Moreover, an antimicrobial effect has been described for (violet-) blue light without the use of exogenous photosensitizers, especially for a wavelength of 405 nm. Multiple studies have indicated that high levels of endogenous porphyrins can also produce reactive oxygen species upon illumination. Hamblin *et al.* stated that *Helicobacter pylori* could be inactivated for 99% after a 405 nm light dose of 30 J/cm^2^. Also *Staphylococcus aureus* and *Pseudomonas aeruginosa* are described to be successfully inactivated by 405 nm light 10,11. Subsequently, a fair amount of studies have investigated the antimicrobial effect of the broader violet-blue wavelengths and demonstrated efficacy in a range of 395 to 470 nm 12-14.

Since this novel intramedullary fracture stabilization system utilizes blue light in the range of 360 - 500 nm for the polymerization and curing of the monomer, there might be a positive side-effect at the surface of the balloon, preventing the formation of a bacterial biofilm. The aim of this study was to assess the inactivation (side-)effect of visible blue light, used in this fracture stabilization system, on *S. aureus*.

## Material and Methods

### Organisms and growth conditions

The strain used in this study was *S. aureus* 8325-4, a well‐established model strain, which has proven to be very important in staphylococcal laboratory research 15. For the experiment an overnight culture on Colombia 5% sheep blood agar (Becton, Dickinson and Company, Sparks, MD, USA) was prepared.

### Intramedullary fracture stabilization system

**Figure 1:**
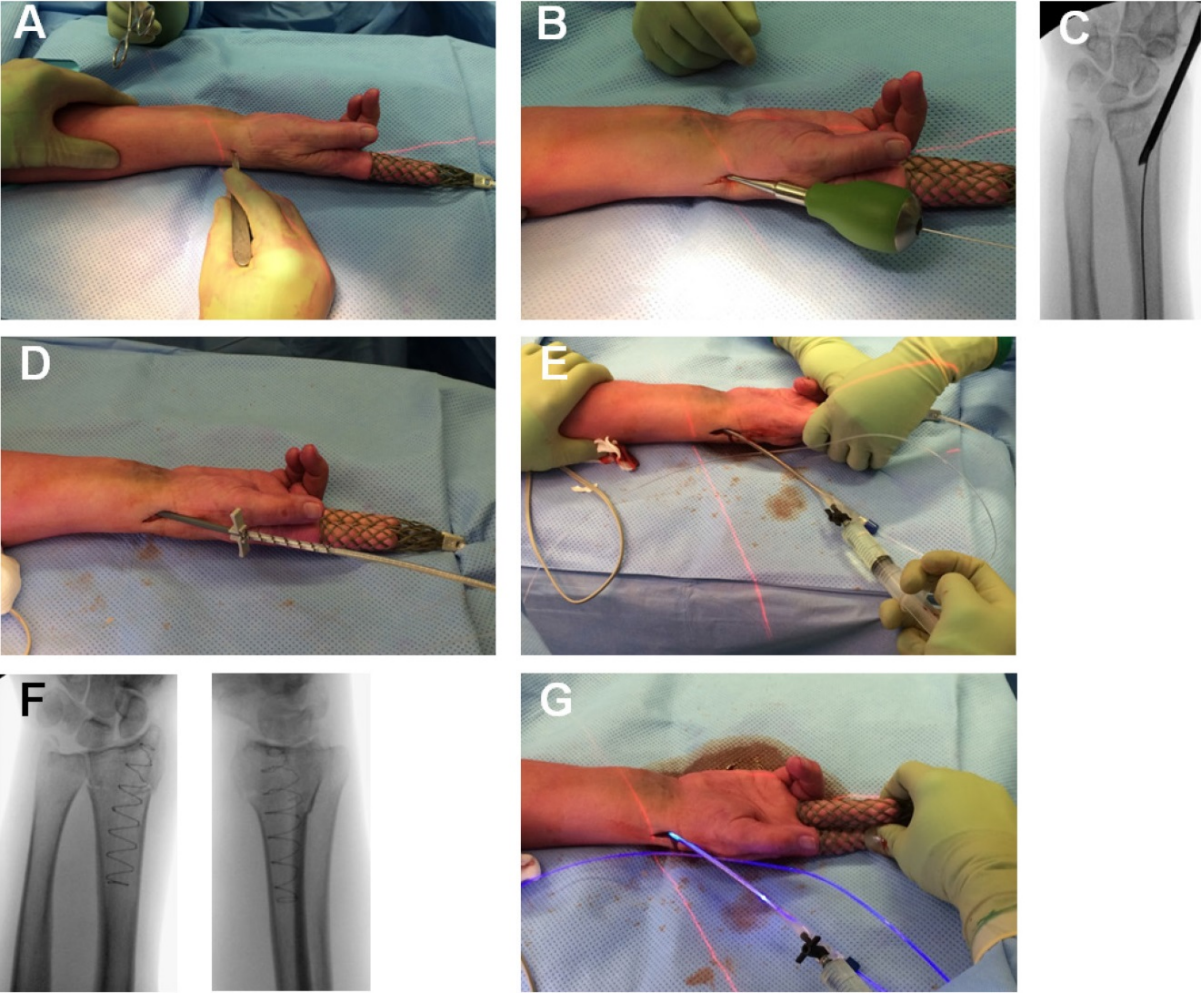
** The intramedullary fracture stabilization system used in a distal radius.** A: Incision over the radial styloid process. B: Access to the medullary canal. C: Correct position is verified by intra-operative fluoroscopy. D: Flexible balloon catheter is placed intramedullary over the guide-wire spanning the fracture. E: Infusion of liquid monomeric material and expansion of the Dacron balloon conforming to the patient's unique medullary canal. F: Verification of adequate fracture reduction, correct balloon position, and balloon expansion. G: Polymerization (hardening) of the infused monomer by applying blue light.

Figure 1 shows the use of the intramedullary fracture stabilization system in a distal radius fracture. It is a minimally invasive intramedullary stabilization system that uses a non-compliant, PET (Dacron) balloon. Over its length, the balloon conforms to the patient's unique medullary canal when it gets filled with liquid monomeric material. This liquid monomer is polymerized (within the balloon) by application of visible blue light via a light catheter for a certain time period. The light source produces light with a frequency-range of 360 - 500 nm in which two intensity peaks, at 405 and 437 nm, are present. The polymerization process takes approximately 200 to 1,000 seconds of blue light exposure, depending on the size of the balloon and, with that, the amount of monomer to be cured. This polymerization process comes with a short peak in temperature after approximately three minutes up till 65 degrees Celsius, and then drops within two minutes to under 37 degrees Celsius 16. In order to secure complete polymerization, the manufacturer supplies a specific time chip with each implant, that should be placed in the standard provided light source. The final result is a patient-specific, intramedullary implant.

### Test set-up

**Figure 2:**
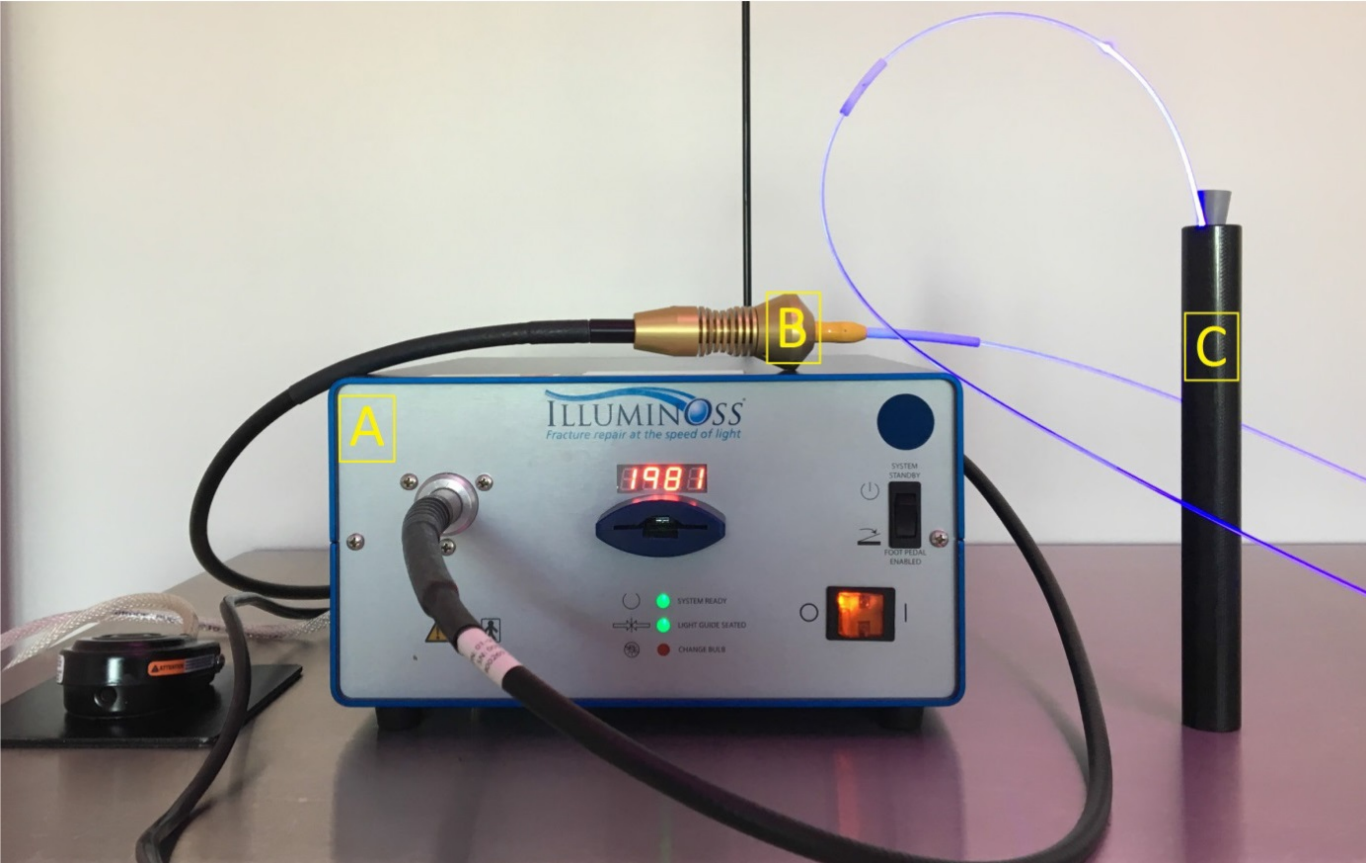
** Test set-up.** A: System light source. B: Light source to light catheter connection. C: Delrin cylinder (simulating bone and intramedullary space) with *S. aureus* suspension and implant getting exposed to blue light.

The test set-up, as shown in Figure 2, was as follows: 250 mL bacterial suspension of 1.5 x 10^3^ Colony Forming Units/mL (CFU/mL) was prepared. Before the start of the first test, a control sample of 100 µL was plated homogeneously on blood agar. Then five, custom made, black delrin cylinders were filled with 7.5 mL of the suspension. Each cylinder had a diameter of 11 mm and a height of 230 mm, and served as a model for the medullary canal. This left 1 mm of space for the suspension around the intramedullary implant, which had a diameter of 9 mm and a length of 180mm. The black delrin material was used to ensure there was no loss of light (Figure 2). Five already polymerized (no polymerization heat) implants were sequentially attached to the light source, which was activated for a period of 200, 400, 600, 800, and 1,000 seconds, and placed in the filled cylinder. The applied light dose was 0.32-0.57 J/cm^2^ after 200 seconds, 0.64-1.15 J/cm^2^ after 400 seconds, 0.96-1.72 J/cm^2^ after 600 seconds, 1.29-2.30 J/cm^2^ after 800 seconds, and 1.60-2.87 J/cm^2^ after 1000 seconds. After the light source was switched off, 100 µL of the suspension was inoculated on a blood agar plate and homogeneously spread. Then all six plates were placed in a 35 degrees Celsius incubator. After 24 hours of incubation, all colonies on each agar plate were counted.

To test for growth on the surface of the implant, after 1,000 seconds exposure time, the implant was taken out of the suspension (with the light still on) and put in a sterile measuring cylinder with Brain Heart Infusion broth (BHI). When the implant was placed in the BHI, the light was switched off, and the measuring cylinder was placed on a vortex for one minute and stirred to suspend any bacteria present on the surface of the implant. The BHI was also incubated at 35 degrees Celsius and was checked on clarity every 24 hours, in order to show bacterial growth. This sequence was repeated five times (test round A - E).

### Data analysis

Both absolute and relative colony reduction, compared with the control plate of the same test round, were calculated in Excel (Microsoft Office Professional Plus 2010). Data were analyzed using IBM SPSS Statistics (Version 24.0). Normality of continuous data was tested with the Shapiro-Wilk test. Homogeneity of variances was tested using the Levene's test. Independent Student's T-tests (with equal variance or unequal variance whichever applied according to the Levene's test) were performed to determine statistical significance of differences in colony count of each experimental group versus the control that was not exposed to light. *P* values <0.05 were considered statistically significant. Spearman's test was used to assess correlations between time and colony count, absolute reduction, and relative reduction.

## Results

### Colony counts

**Figure 3:**
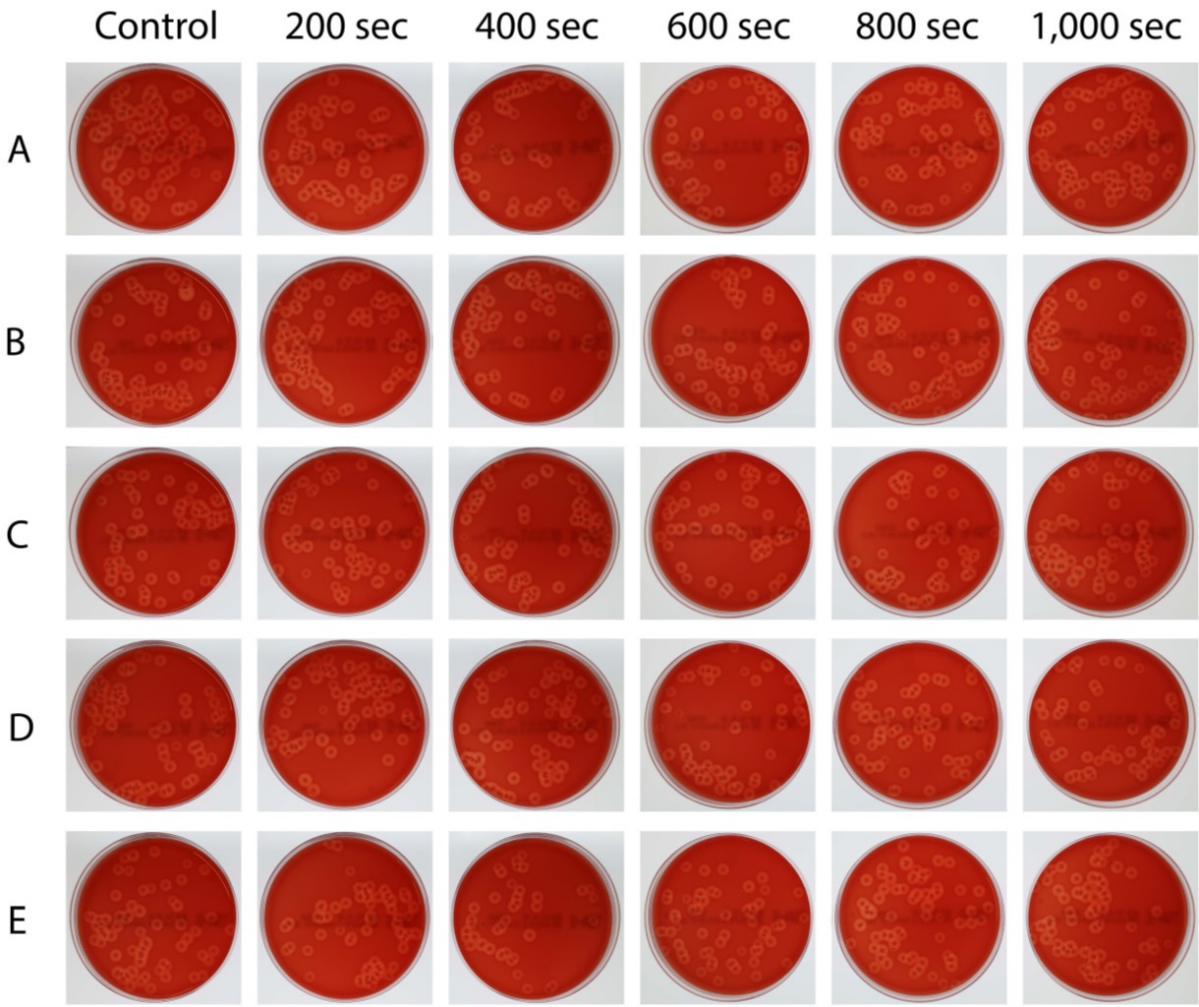
** Overview of study results.** Individual petri dishes with colonies for the five different light exposure times as well as the unexposed control are shown for measurement series A to E.

**Figure 4:**
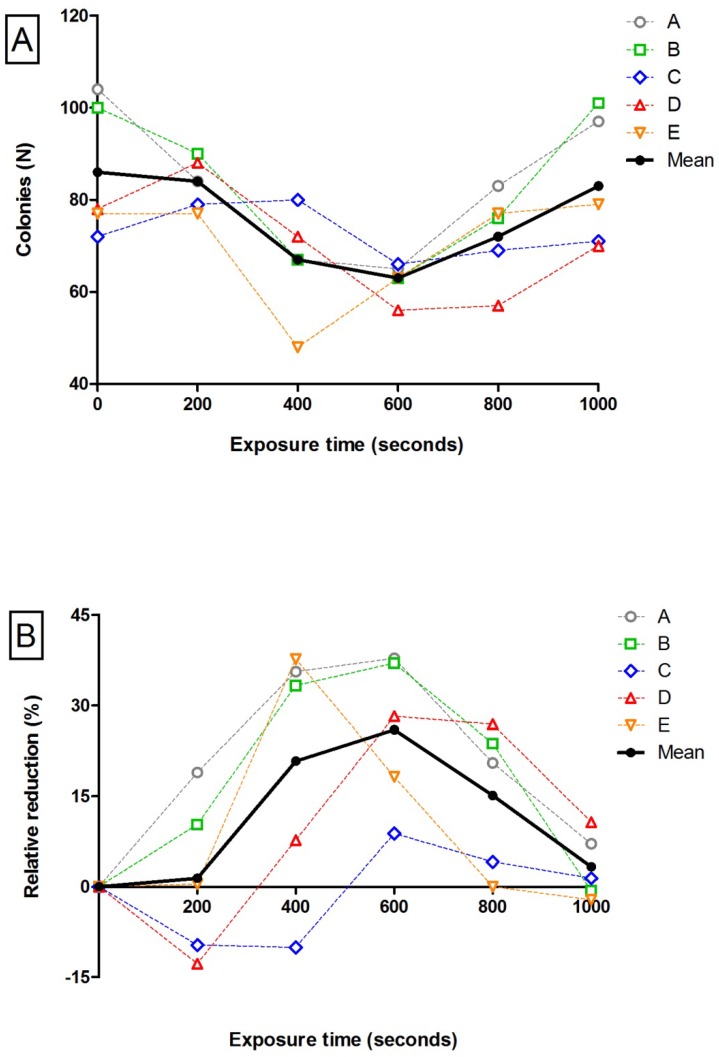
** Colony counts and colony reductions per exposure time.** A: Colony counts for control plates and after the five exposure times. B: Relative reduction rates compared with control plates for the five exposure times. Data are shown as N. The black line shows the average of the measurements in series A to E.

Figure 3 shows an overview of all agar plates. The control plates where no blue light was applied to the bacterial suspension, showed a mean of 85 ± 15 colonies per plate. A statistically significant decrease was observed after 600 seconds of exposure time; the mean colony count was 63 ± 4 colonies per plate (p <0.05) as shown in Table 1. The absolute reduction was 24 colonies ± 14 after the 600 seconds exposure time. At 800 and 1,000 sec, on the other hand, no statistically significant reduction was found compared with the control plates (means 72 ± 10 and 83 ± 14 colonies, respectively). The highest mean relative reduction was again observed after 600 seconds (26% reduction), where only a 3% relative reduction was seen after 1,000 seconds (Table 2). No statistically significant correlation was found between time and the number of colonies (Pearson correlation coefficient -0.229, p=0.223). Figure 4 displays the colony counts, absolute reduction and relative reduction rates for all five test series (A-E).

### Brain Heart Infusion broth

In all five test series, the BHI was unclear after 24 hours in a 35 degrees Celsius blood stove, indicative for bacterial growth on the surface of the implant, despite 1000 seconds of blue light exposure.

## Discussion

The aim of this study was to assess a potential bactericidal effect of visible blue light, used in a novel intramedullary fracture stabilization system, on *S. aureus*. If present, it would be a favorable side effect of this system, which is primarily designed to stabilize bone fractures. The experimental design simulated the clinical use of the fracture stabilization system as good as possible. Although a statistically significant decrease in colony count was observed after 600 seconds of blue light, this effect had surprisingly disappeared after 800 seconds and 1,000 seconds exposure time.

This hyperbolic effect over time is in contrast to what has been described in literature. A recent systematic review aimed to compile the current knowledge on the antimicrobial efficacy of violet-blue light (380-480 nm) and concluded that additional research is required to ensure whether this technology is effective. Moreover, the exact mechanism of inactivation and the potential for bacterial tolerance is unclear 17. Despite the absence of this knowledge several studies showed high inactivation rates, up to 99% by visible light, for various bacterial species 8,10,11. The current study did not reproduce these high inactivation rates.

From literature it is known that the inactivation of bacteria by visible light consists of three cornerstones: 1) an adequate light dose in the appropriate wave length, 2) endo- or exogenous porphyrins, 3) oxygen 18.

In this study, no correlation was observed between the time of light exposure and a bactericidal effect. The hyperbolic results in colony counts and the absence of a bactericidal effect after the highest light dose was applied, make it highly unlikely that an inadequate light dose is the cause for our hyperbolic results in colony counts over time. However, we cannot exclude that the total light dose or the manner of application is inadequate. For example, the polymer or Dacron material might block certain light frequencies, however the statistically significant reduction after 600 seconds implies adequate light frequency. In order to assess the adequacy of the light dose, a higher dose (i.e. higher intensity radiance or longer exposure time) without interfering of the implant, can be applied. But such a set up would not reflect the clinical use of the fracture stabilization system.

The possible lack of oxygen in our test set-up, might be another explanation for the hyperbolic results in colony counts over time. Since the suspension was used in a closed cylinder without any oxygen enhancement, a relative oxygen depletion might have been present after 800 and 1,000 seconds. Moreover, it is known that *S. aureus* is able to grow without oxygen. With this possible oxygen depletion, no new reactive oxygen species can be formed, thus no inactivation of bacteria can be achieved. As this fracture stabilization system is used intramedullary with continuous supply of oxygen, this depletion might not be an issue in a clinical setting. If desired, additional research could be performed to assess whether a lack of oxygen is actually the reason for the low inactivation rates of *S. aureus*.

## Conclusion

Despite the fact that previous studies with different experimental designs described an antimicrobial effect of visible blue light, and the manufacturer's belief in an antimicrobial effect of the fracture stabilization system, this study only showed a temporary reduction in bacterial count of *S. aureus.* For clinical relevance (*i.e.* less infections) a sustained reduction will be essential. If future research regarding the antimicrobial characteristics of blue light used in the IlluminOss^®^ system is desired, it should focus on the need for and availability of oxygen, and the dose and manner of applying the light.

## Figures and Tables

**Table 1:** Colony counts per test sequence

	Control (0 sec)	200 sec	400 sec	600 sec	800 sec	1,000 sec
A	104	84	67	65	83	97
B	100	90	67	63	76	101
C	72	79	80	66	69	71
D	78	88	72	56	57	70
E	77	77	48	63	77	79
Mean (SD)	86 (15)	84 (6)	67 (12)	63 (4)	72 (10)	83 (14)

The absolute number of colonies per agar plate is shown for test series A to E.

**Table 2:** Relative colony reduction per test sequence

	200 sec	400 sec	600 sec	800 sec	1000 sec
A	18.9	35.6	37.8	20.5	7.1
B	10.3	33.3	37.0	23.7	-0.7
C	-9.7	-10.1	8.8	4.1	1.4
D	-12.8	7.7	28.2	26.9	10.7
E	0.4	37.7	18.2	0.0	-2.2
Mean (SD)	1.4 (13.3)	20.8 (21.1)	26.0 (12.5)	15.1 (12.1)	3.3 (5.4)

Data are shown as %, calculated from control

## Abbreviations

PET: Polyethylene terephthalate
ROS: Reactive oxygen species
CFU: Colony Forming Units
BHI: Brain Heart Infusion
